# Phylogenetic and Autecology Characteristics of Five Potentially Harmful Dinoflagellate *Alexandrium* Species (Dinophyceae, Gonyaulacales, Pyrocystaceae) in Tropical Waters: *A. affine*, *A. fraterculus*, *A. leei*, *A. pseudogonyaulax*, and *A. tamiyavanichii*

**DOI:** 10.3390/toxins17020081

**Published:** 2025-02-10

**Authors:** Lam Nguyen-Ngoc, Dang-Minh Luat, H. Doan-Nhu, H. M. Pham, B. Krock, N. D. Huynh-Thi, L. V. Tran-Thi, M. H. Tran-Thi, Anh H. Pham, V. Nguyen-Tam, T. T. Nhan-Luu, H. H. Do

**Affiliations:** 1 Institute of Oceanography, VAST, 01 Cau Da Str., Vinh Nguyen Ward, Nha Trang City 650000, Vietnam; haidoan-ion@planktonviet.org.vn (H.D.-N.); duyenhuynh@planktonviet.com (N.D.H.-T.); levan1981vhdh@gmail.com (L.V.T.-T.); minhhuehdg@yahoo.com (M.H.T.-T.); vinh.nguyen0512@gmail.com (V.N.-T.); dohuuhoang2002@yahoo.com (H.H.D.); 2 Department of Ecology and Evolutionary Biology, University of Science, 227 Nguyen Van Cu Str., 5th Dist., Ho Chi Minh Chity 700000, Vietnam; dangmluat@gmail.com (D.-M.L.); lttnhan@hcmus.edu.vn (T.T.N.-L.); 3 Vietnam National University, Ho Chi Minh City 700000, Vietnam; 4 Alfred-Wegener-Institut für Polar- und Meeresforschung Ökologische, Chemie Am Handelshafen 12, 27570 Bremerhaven, Germany; 5 Biology Department, Woods Hole Oceanographic Institution, Woods Hole, Falmouth, MA 02543, USA; anh.pham@whoi.edu

**Keywords:** *Alexandrium* spp., ITS, LSU (D1–D3), LSU (D8–D10), growth rate, PSTs, Vietnam

## Abstract

Five species of *Alexandrium* (*A. affine*, *A*. *fraterculus*, *A*. *leei*, *A*. *pseudogonyaulax,* and *A*. *tamiyavanichii*) are commonly found in Vietnamese waters. They were distinguished based on their apical pore complex (A.P.C), precingular first plate (1′), ventral pore (Vp), and sulcal platelets. A genetic analysis was conducted using nuclear rDNA sequences of ITS and LSU (D1–D3, D8–D10). The growth rates of *A*. *fraterculus*, *A*. *leei*, *A*. *tamiyavanichii*, and *A*. *pseudogonyaulax* were quite similar. Specifically, these four species had the highest growth rates at two temperature levels of 24 °C and 27 °C, at salinities ranging from 25 psu to 35 psu. Furthermore, these species were able to adapt to a low salinity of 20 psu at temperatures from 18 °C to 27 °C. No Paralytic Shellfish Toxins (PSTs) were found in the two *Alexandrium affine* strains, VINVN01-1 and VINVN01-2. The detection limit for PSTs ranged from 0.45 to 15.5 fg cell^−1^, depending on the molecular response and available biomass.

## 1. Introduction

Thirty-three species of *Alexandrium* have been found to date [1]. *Alexandrium* Halim is common in marine and brackish waters [2,3,4,5]. Among all *Alexandrium* species, 14 are known to be toxin producers, causing Paralytic Shellfish Poisoning (PSP); outbreaks have negative impacts on marine ecosystems, the seafood industry, and tourism, causing deaths among marine animals and leading to sickness and fatalities in humans via the consumption of contaminated seafood [3,6]. Hence, the population dynamics and toxin production of the *Alaxandrium* species are major concerns for scientists, farmers, and governments.

Twenty-five people from Korea have been poisoned by Paralytic Shellfish Toxins (PSTs), with two deaths reported after consuming wild mussels [7]. Significant blooms of the potentially toxic Alexandrium affine were recorded from Ambon Bay, Indonesia, but no harmful effects or damage to local fisheries were reported [8,9]. Alexandrium spp. have bloomed in Thailand [10], and six *Alexandrium* species (*A*. *cohorticula*, *A*. *fraterculus*, *A*. *leei*, *A*. *tamarense*, *A*. *tamiyavanichi*, and *A. minutum*) were found [11,12]. A. tamiyavanichi was found to be a producer of PSTs, while A. tamarense was found to be non-toxic [13,14]. However, the cell densities of these species were frequently low, and there have been no reports discussing the impact of PSTs on human health [12]. Recently, *Alexandrium fragae, A*. *pohangense, A*. *pseudogonyaulax, and A*. *tamutum* were found in the Gulf of Thailand [15]. Four tropical PSP toxins-producing dinoflagellates, *Alexandrium minutum, A. tamiyavanichii, A. tamarense,* and *A. peruvianum* from Malaysian waters were studied to investigate the influences of salinity on growth and toxin production [16]. Cultured isolates of *A. minutum* and A. *tamiyavanichi* produced PSTs, but *A*. *tamarense* clones were not found to be toxic [16]. An *Alexandrium minutum* bloom was first reported in the Sungai Geting lagoon in Malaysia [17], but harmful effects were not detected. Two new species, *Alexandrium limii* sp. nov. S.T.Teng, Tillmann, N. Abdullah, and S. Nagai et Leaw, and *A*. *ogatae* sp. nov. S.T. Teng, N. Abdullah, Tillmann, and Leaw et P.T. Lim, are known to produce goniodomins, but no human intoxication has been reported in Southeast Asia [18].

Over the past two decades, the species diversity of *Alexandrium* in Vietnamese waters has been extensively studied, with 14 species initially being identified and described [19]. Subsequently, *Alexandrium satoanum* was found in upwelling waters in Phan Ri, Tuy Phong Bay (Binh Thuan Province), while *A*. *tamutum* was reported in shrimp ponds with salinities ranging from 25 [20] to as low as 5 [21]. A restricted distribution of *Alexandrium foedum* was reported in Lang Co Bay, Thua Thien Hue [22]. Thus far, a total of 17 *Alexandrium* species have been documented in Vietnamese waters (Table 1). The majority of *Alexandrium* species in Viet Nam have not undergone toxin testing, with the exception of *A. affine*, sampled from Ha Long Bay (North Vietnam), and *A*. *leei*, collected from Nha Phu estuary (Khanh Hoa Province), which were found to contain NeoSTX, STX, and GTX1-4 at concentrations below 1.0 fmol per cell [23]. In recent decades, there has been increasing research on *Alexandrium’s* morphology and ability to produce toxins [24,25,26,27]. The integration of molecular-based phylogenetic information into the morphologic approach has become essential in identifying and distinguishing highly similar *Alexandrium* species, as well as in studying their distributions and evolution [28,29].

The genus *Alexandrium* was split into four genera, *Alexandrium*, *Gessnerium*, *Protogonyaulax*, and *Episemicolon*, with the latter being a new genus [30]. However, this division was disputed [31], and currently only one accepted genus of *Alexandrium*, with 34 recognized species, exists [1].

The growth of *Alexandrium*, including the initiation and development of *Alexandrium* blooms, can be driven by salinity, temperature, and water-column stratification [32]. The growth rates of *Alexandrium* species were found to vary with different nutrient conditions, where nitrogen and phosphorus play vital roles [33,34,35,36]; the effects of salinity and temperature on the growth rate of *A. affine* isolated from Ha Long Bay were also documented [22].

Five species of *Alexandrium* are commonly observed in Nha Trang Bay, including *A. affine, A*. *fraterculus, A*. *pseudogonyaulax, A*. *leei,* and *A*. *tamiyavanichii.* These species were morphologically described by Nguyen-Ngoc and Larsen [19], and in the present study, we briefly focus on their morphological characteristics. The first three species were independently analyzed for their genetic characteristics using molecular data, specifically three rDNA regions: D1–D3, D8–D10, and ITS1-5.8S-ITS2. Given the frequent presence of *A. affine* along the Vietnamese coast and the aforementioned observations, we analyzed the toxicity of *A. affine* from Nha Trang Bay to reconfirm its toxic potential. Finally, autecological experiments were conducted on *A*. *fraterculus*, *A*. *leei*, *A. pseudogonyaulax*, and *A*. *tamiyavanichii* to gather information on their growth rates and their capacity to form harmful blooms under the temperature and salinity of tropical waters.

## 2. Results

*Alexandrium affine*, *A*. *fraterculus*, and *A*. *tamiyavanichii* were found in the months from June to August in waters with salinities of 27–28 psu and temperatures of 27.5 °C–29 °C. *A*. *leei and A*. *pseudogonyaulax* were isolated from coastal surface waters at a depth of 1.5–3 m, temperature of 29 °C, and salinity of 30 psu. Profiles of temperature and salinity in Nha Trang Bay was resulted in Figure S1.

### 2.1. Identifying Alexandrium Species

#### 2.1.1. Morphological Observations

##### *Alexandrium affine* (Figure 1A–D)

**Figure 1:**
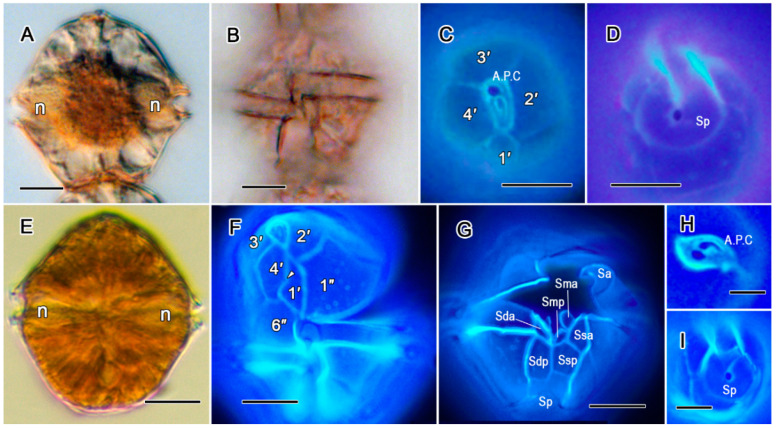
(**A**–**I**). *Alexandrium* ((**A**,**B**,**E**): D.I.C.; (**C**,**D**,**F**,**G**–**I**): epifluorescent).—*Alexandrium affine* (**A**–**D**): (**A**) ventral view of cell, showing the location of the nucleus (n); (**B**) ventral view of cell, showing the cingulum descending; (**C**) apical view, showing plate pattern; (**D**) antapical view, showing V-shaped posterior sulcal plate with large connecting pore. *Alexandrium fraterculus* (**E**–**I**): (**E**) ventral view of cell, showing full chloroplast and location of nucleus (n); (**F**,**G**) ventral view of squash cells, showing plate pattern of epitheca and sulcal plates of hypotheca; (**H**,**I**) the tetragonal apical pore plate; (**I**) V-shaped posterior sulcal plate with large antapical connecting pore. All scale bars = 10 µm.

Cells were almost isodiametric in size and convex–pentagonal in shape (Figure 1A). The cingulum descending, displaced about one girdle width (Figure 1B). The apical pore complex (A.P.C) had a large connecting pore located dorsally relative to the comma-shaped apical pore (Figure 1C). The posterior sulcal plate (S.p.) was V-shaped and had an attachment pore (Figure 1D).

Molecular data: strain VINVN01: PP106429 (D1–D3, LSU rDNA), PP106432 (D8–D10, LSU rDNA), and PP106377 (ITS1-5.8S-ITS2).

##### *Alexandrium fraterculus* (Figure 1E–I)

Cells were pentagonal and isodiametric in size (Figure 1E). The apical pore complex (A.P.C.) was a convex tetragonal plate with a connecting pore (Figure 1H). A ventral pore was located one-third of way along the posterior right margin in the first apical plate (1′, Figure 1F). Sulcal platelets were observed (Figure 1G). The sulcal posterior plate (S.p.), which had a V-shaped sulcus-directed border, was isodiametric with a centrally located connecting pore (Figure 1I).

Molecular data: strain VINVN02: PP106430 (D1–D3), PP106433 (D8–D10), and PP106378 (ITS1-5.8S-ITS2).

##### *Alexandrium leei* (Figure 2A–D)

**Figure 2:**
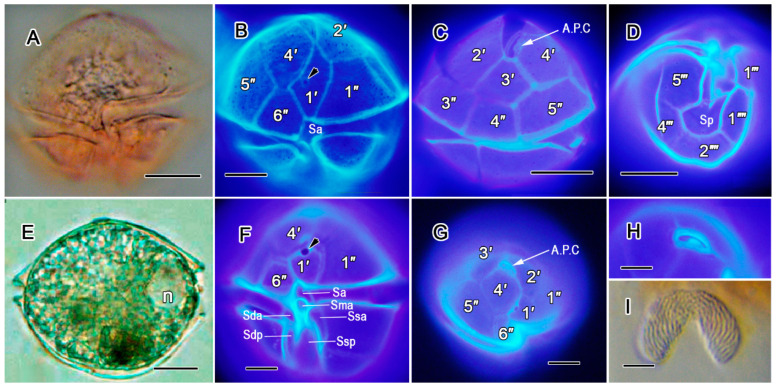
*Alexandrium leei* (**A**–**D**): (**A**) ventral view of cell; (**B**) ventral view of cell, showing plate pattern and 1′-plate with small ventral pore (arrow head); (**C**) apical dorsal view; (**D**) antapical ventral view showing plate pattern. *Alexandrium pseudogonyaulax* (**E**–**I**): (**E**) ventral view of cell with nucleus (n) position; (**F**) ventral view of cell, showing plate pattern an a large ventral pore (arrow head) on 1′; (**G**) part of the apical view with plate pattern of A.P.C; (**H**) pore plate with A.P.C. at high magnification; (I) the U-shaped nucleus. ((**B**–**D**,**F**–**H**): epifluorescent; (**A**,**E**,**I**): D.I.C.). All scale bars = 10 µm.

Cell size was somewhat isodiametric, with the length being equal to a width of approximately 35 μm. The apical pore complex was narrow and markedly angular, without an attachment pore, but had a comma-shaped pore (Figure 2C). The ventral pore was small, located inside the 1′ plate, and situated near the right margin of the plate (Figure 2B). The cingulum had a descending replacement (Figure 2A,B). The posterior sulcal plate (Sp) was small and had no connecting pores (Figure 2D).

Molecular data: not available.

##### *Alexandrium pseudogonyaulax* (Figure 2E–I)

Cell was irregularly pentagonal in shape (Figure 2E). The first apical plate (1′) had a briefly truncated pentagonal shape (Figure 2F). Thus, the 1′ plate completely disconnected from the apical pore complex, which only had a comma, without the connecting pore (Figure 2G,H). A large ventral pore appeared at the junction of the 1′ plate and the 4′ plate (Figure 2F,G). The sulcus was large with clear platelets (Figure 2F). The nucleus was of a U-shape (Figure 2E,I).

Molecular data: strain VINVN003: PP106431 (D1–D3), PP106434 (D8–D10), and PP106379 (ITS1-5.8S-ITS2).

##### *Alexandrium tamiyavanichii* (Figure 3A–F)

**Figure 3:**
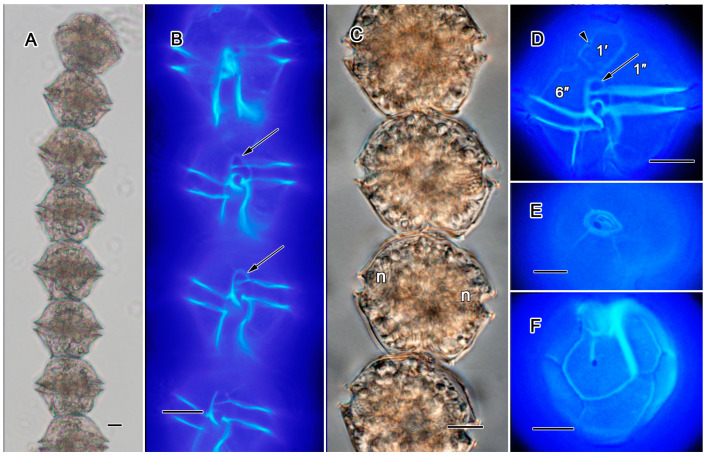
(**A**–**F**)**.** *Alexandrium tamiyavanichii*: (**A**,**B**) cells connected in a chain, showing precingular part on the anterior sulcal plate (arrows and Figure 1D) and nucleus (n) position; (**D**) ventral view of cell, showing 1′-plate with a tiny ventral pore (a head arrow); (**E**) apical pore complex with a large connecting pore; (**F**) antapical view showing the posterior sulcal plate with a large connecting pore. All scale bars = 10 µm.

Cells often formed long chains of up 32 cells (Figure 3A,B, Supplementary Video S1), with each cell having a length 38–50 µm and width 40–50 µm. The nucleus was U-shaped (Figure 1C). The first apical plate (1′) was directly attached to the APC; both plates were arranged obliquely to each other on a horizontal plane. A small ventral pore was located on the right margin of the 1′ plate (Figure 1D). The anterior sulcal plate had a precingular part that was dome-shaped (Figure 1B,D). The sulcal posterior plate had a V-shaped sulcus-directed border and a centrally located posterior attachment pore (Figure 1F), connected to a narrow groove in the anterior portion of the right margin (Figure 1F).

Molecular data: not available.

#### 2.1.2. Phylogenetic Analyses

**Figure 4:**
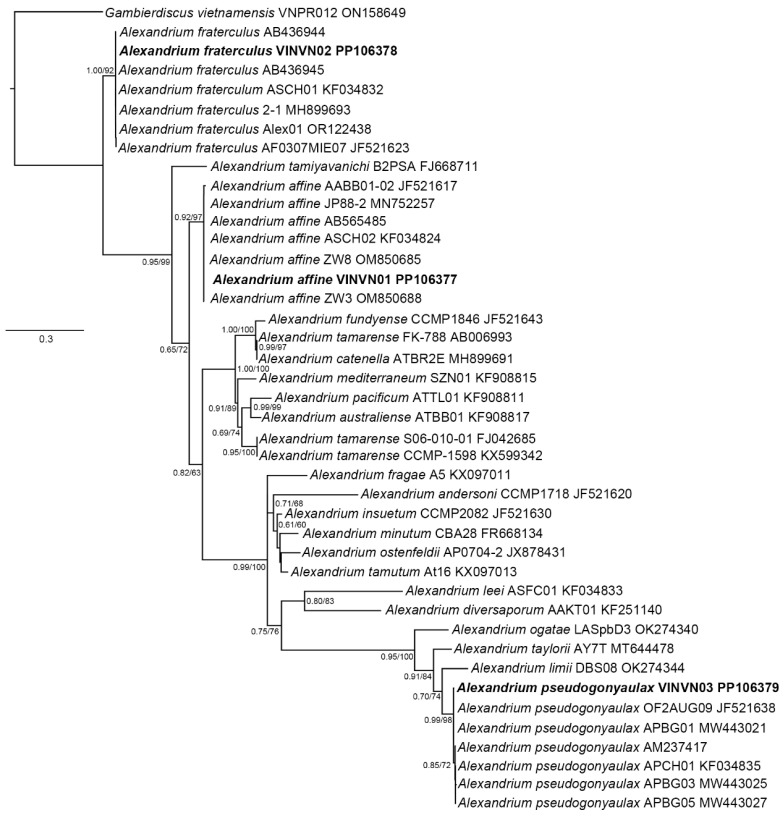
Maximum likelihood phylogeny of the ITS1-5.8S-ITS2 region for *Alexandrium* spp. and *Gambierdiscus vietnamensis* (outgroup). Bayesian posterior probability (**left**) and the bootstrap values (**right**) shown in each node; names of species observed in this study are printed in bold.

**Figure 5:**
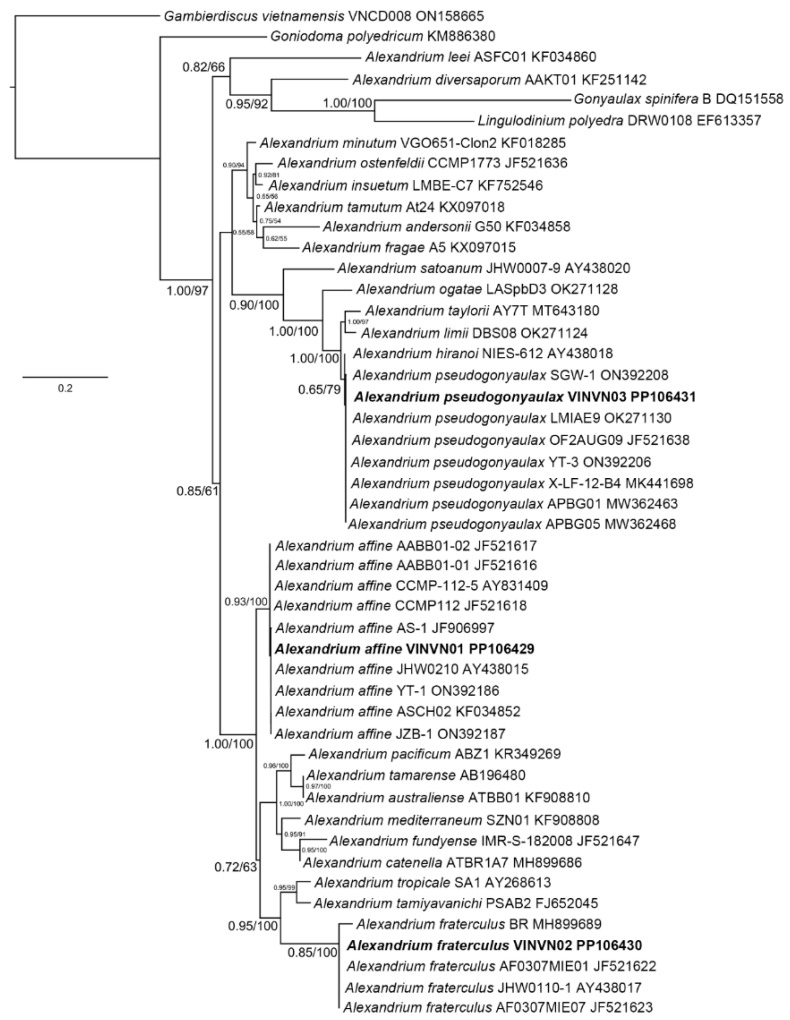
Maximum likelihood phylogeny of the D1–D3 rDNA region for *Alexandrium* spp., *Goniodoma polyedricum*, *Gonyaulax spinifera*, *Lingulodinium polyedra*, and *Gambierdiscus vietnamensis* (outgroup). Bayesian posterior probability (**left**) and the bootstrap values (**right**) are shown in each node; the names of species observed in this study are printed in bold.

**Figure 6:**
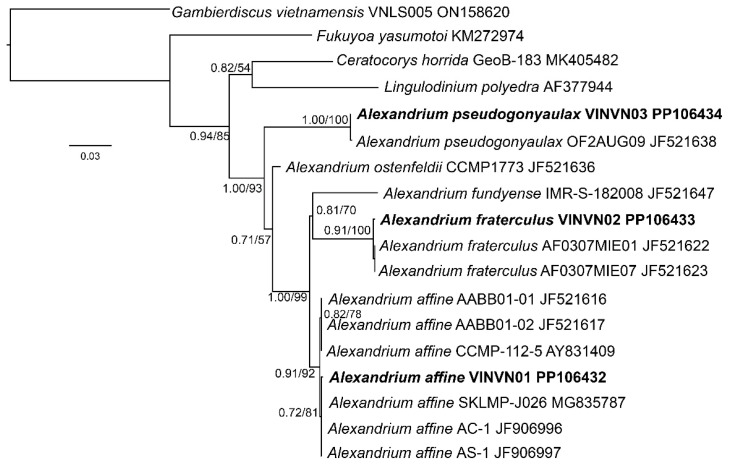
Maximum likelihood phylogeny of the D8–D10 region for *Alexandrium* spp., *Fukuyoa yasumotoi*, *Ceratocorys horrida*, *Lingulodinium polyedra*, and *Gambierdiscus vietnamensis* (outgroup). Bayesian posterior probability (**left**) and the bootstrap values (**right**) are shown in each node; names of species observed in this study are printed in bold.

A multiple alignment, including gaps, was generated for 42 taxa (ITS1-5.8S-ITS2), 48 taxa (D1–D3), and 18 taxa (D8–D10) (Table 2). The phylogenetic reconstructions obtained from these datasets are presented in Figure 4, Figure 5 and Figure 6. The ML and BI analyses based on the ITS1-5.8S-ITS2, D1–D3, and D8–D10 regions resulted in phylogenetic trees with similar topologies. The results of the two algorithms showed that the sequences of *Alexandrium* spp. (VINVN01, VINVN02, VINVN03) collected in Vietnam matched those of *A. affine*, *A*. *fraterculus* and *A*. *pseudogonyaulax*, respectively, with *p*-distances < 0.01. The bootstrap support values of ML and the posterior probability value of BI were high for the nodes of these species, at >92% and >0.85, respectively (Figure 4, Figure 5 and Figure 6). *Alexandrium affine*, *A*. *fraterculus,* and *A*. *pseudogonyaulax* were monophyletic and distributed into three different clades (Figure 4, Figure 5 and Figure 6).

### 2.2. Effects of Temperature and Salinity on Growth of Alexandrium Species

#### 2.2.1. *Alexandrium fraterculus*

**Figure 7:**
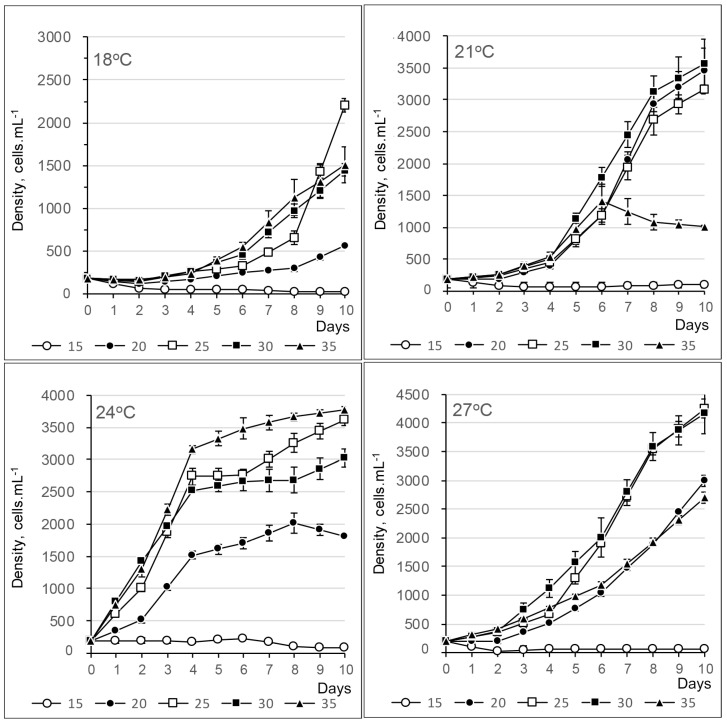
Growth curves of *Alexandrium fraterculus* at different temperatures and salinities.

The growth of *Alexandrium fraterculus* was generally slow at 18 °C, with growth rates ranging from 0.4 to 0.6 divisions per day across all salinities. Cell densities reached around 1.5 to 2 × 10^3^ cells mL⁻^1^ at salinities between 25 psu and 35 psu by the 10th day (Figure 7 and Figure S2). An exponential phase was observed from the 4th day of treatment at 21 °C, reaching around 3–3.5 × 10^3^ cell mL^−1^ at salinities from 20 psu to 30 psu on the 10th day. At 24 °C, cell divided quickly (0.8–1.0 divisions day^−1^) from the beginning until the 4th day, then slightly increased to 3–3.5 × 10^3^ cell mL^−1^ at salinities from 25 to 35 psu on the 10th day. At 27 °C, cells slowly reached the exponential phases on the 4th day and continued to increase to around 4 × 10^3^ cell mL^−1^ at salinities of 20 and 30 psu on the 10th day.

**Figure 8:**
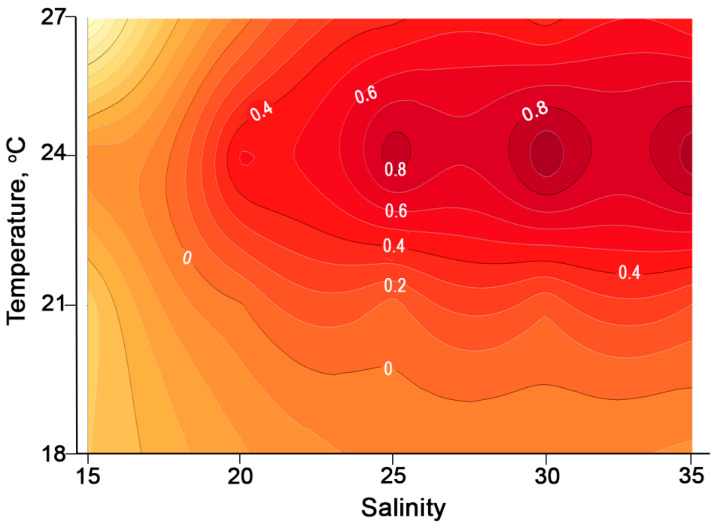
Contour plot of specific growth rate (day^−1^) of *Alexandrium fraterculus* as a function of temperature and salinity.

Growth rates generally increased with an increase in temperatures from 18 °C to 24 °C, regardless of salinity (Figure 8). Repeated measures ANOVA (*p* < 0.05) and Tukey post hoc tests (*p* < 0.05) indicated that most growth rates across different temperature and salinity combinations were significantly different. At a salinity of 15 psu, growth rates were mostly negative or near zero across all temperature groups (Figure 8).

#### 2.2.2. *Alexandrium tamiyavanichii*

**Figure 9:**
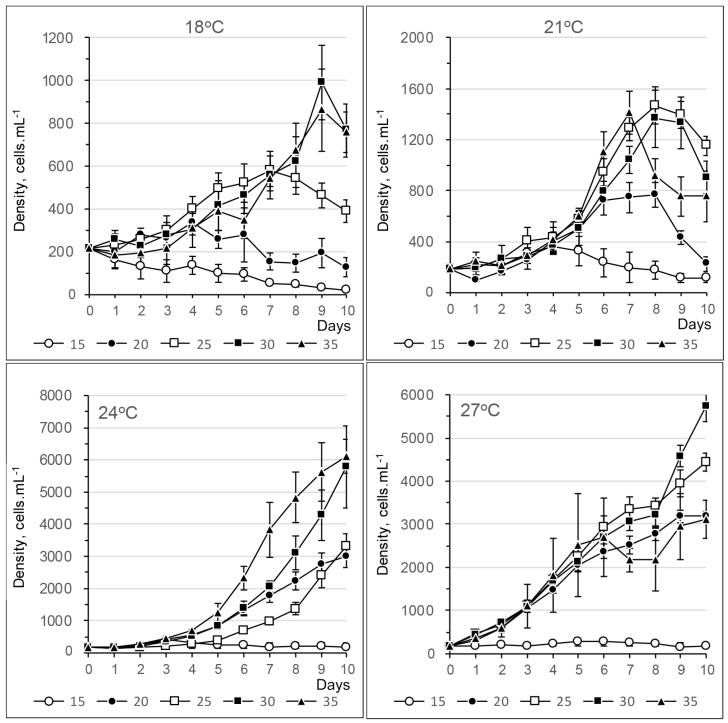
Growth curves of *Alexandrium tamiyavanichii* at different temperatures and salinities.

**Figure 10:**
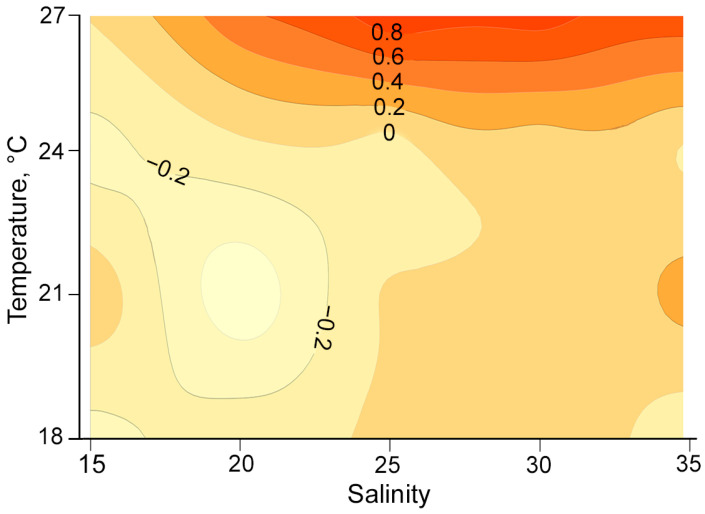
Contour plot of specific growth rate (day^−1^) of *Alexandrium tamiyavanichii* as a function of temperature and salinity.

At 18 and 21 °C, the growth of *Alexandrium tamiyavanichii* was variable but generally slow, with growth rates ranging from 0.2 to 0.6 divisions per day. Cell densities did not exceed 1.6 × 10^3^ cells mL⁻^1^ after 9 days (Figure 9). At 24 °C, exponential growth phases were observed from the 5th day, with growth rates of between 0.4 and 0.6 divisions per day. Cell densities reached approximately 6 × 10^3^ cells mL⁻^1^ at salinities of 30 and 35 psu by the 10th day (Figure 10). At 27 °C, cells divided rapidly on the first day (0.6 to 1 divisions per day), followed by a gradual decline in growth rates. Nevertheless, the cell density continued to rise, surpassing 5.5 × 10^3^ cells mL⁻^1^ at a salinity of 30 psu by the 10th day. Growth rates were below 0.2 divisions day^−1^ and mostly negative for all temperatures at a salinity of 15 psu (Figure S3).

#### 2.2.3. *Alexandrium leei*

**Figure 11:**
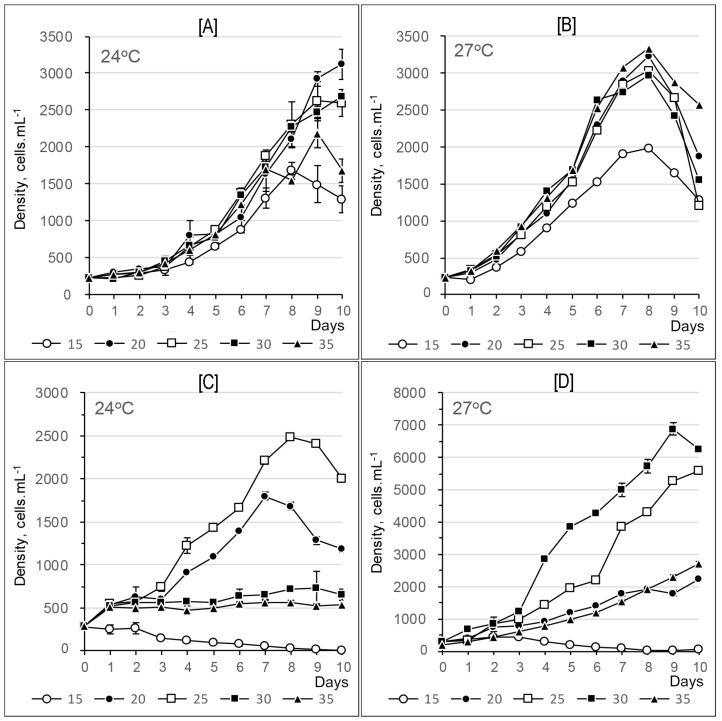
Growth curves of *Alexandrium leei* (**A**,**B**) and *A*. *pseudogonyaulax* (**C**,**D**) at different salinities and temperatures of 24 °C and 27 °C.

The growth of *Alexandrium leei* exhibited sustained upward trends from day 1 to around day 8, followed by slight decreases, most notably at 27 °C (Figure 11A,B). Salinity treatments between 20 and 35 psu yielded maximum cell densities of approximately 2–2.3 × 10^3^ cells mL⁻^1^ at 24 °C and 3–3.2 × 10^3^ cells mL⁻^1^ at 27 °C. In contrast, a salinity of 15 psu resulted in cell densities consistently below 2 × 10^3^ cells mL⁻^1^ throughout the experiment at both temperatures.

Growth rates ranged from 0.22 to 1.06 divisions per day at 24 °C and from 0.11 to 0.63 divisions per day at 27 °C (Table 3), with the highest rates observed on the fourth day of the experiment. Repeated measures ANOVA indicated that temperature alone did not have a significant effect on the growth rate of *Alexandrium leei* (*p* > 0.05). However, salinity was identified as a significant factor influencing growth rate (*p* < 0.05).

#### 2.2.4. *Alexandrium pseudogonyaulax*

The growth of *Alexandrium pseudogonyaulax* varied significantly across different salinity and temperature combinations. At 24 °C, the exponential growth phase began on day 4, with a growth rate ranging from 0.4 to 0.5 divisions per day, and continued until the 7th day at salinities of 20 psu and 25 psu (Figure 11C,D, Table 4). The cell density peaked at approximately 2.5 × 10^3^ cells mL⁻^1^ at a salinity of 25 psu on the 8th day, followed by a notable decline.

At 27 °C, the exponential phase also started on day 4, with cell densities reaching between 5.5 and 7 × 10^3^ cells mL⁻^1^ at salinities of 25 and 30 psu by the 10th day (Figure 11, Table 4). The growth rate peaked at from 0.6 to 1 division per day on different days, depending on salinity, which ranged from 20 psu to 35 psu. In contrast, the cell division rate was predominantly negative at a salinity of 15 psu for both temperature conditions (Table 4).

Repeated measures ANOVA and subsequent post hoc tests confirmed that both temperature and salinity had significant effects on the growth rate of A. pseudogonyaulax (*p* < 0.05).

### 2.3. Paralytic Shellfish Toxins

No PSTs were detected in the two *Alexandrium affine* strains. The detection limit was between 0.45 and 15.5 fg cell^−1^ depending on the molecular response of the individual PSTs and the available biomass. The detailed detection limits for each PST and strain are shown in Table 5.

## 3. Discussion

### 3.1. Identifying Alexandrium Species

#### 3.1.1. Morphological Observations

*Alexandrium* species collected in the south–central coastal waters of Vietnam were distinguished via their morphological characteristics, including their cell size and shape, plate pattern, ventral pore presence and position, and anterior and posterior attachment pore locations. However, these traits vary widely and can overlap or slightly transition between species, leading to potential discrepancies in morphological identification in descriptive documents [2,37]. The three species *A. affine*, *A*. *fraterculus*, and *A*. *tamiyavanichi* all have a roughly similar size and a similar morphology of the 1′, 6”, S.a. and S.p. plates; however, the S.a. plate of *A*. *tamiyavanichi* has a precingular part [19,38] and this characteristic is not found in the other two species. *A. affine, A*. *fraterculus,* and *A*. *tamiyavanichi* can form a long chain of up to 32 cells (Supplementary Video Clip S1) with a wide distribution, which is commonly found in tropical waters of South East Asia (SEA). Three two species differ markedly in the shape of the apical pore plate and the position of the apical connecting pore (Figure 1C,H), which is morphologically consistent with the descriptions [19,38,39,40].

#### 3.1.2. Phylogenetic Data

The ITS1–5.8S-ITS2 rDNA regions and D8–D10 domains of LSU were useful for identifying *Alexandrium* spp., showing substantial distinctions between species. Among the three species recorded in Vietnam, *A. affine* and *A*. *fraterculus* are closely related to each other, as shown in Figure 4 and Figure 5, whereas *A*. *pseudogonyaulax* is separated from these two species and belonged to the clade of *A*. *limii, A. taylorii,* and *A*. *ogatae* (Figure 4). This is similar to the phylogenetic findings of Abdullah et al. (2023) [25]. In the D1–D3 phylogeny (Figure 3), there was no genetic difference between *A*. *pseudogonyaulax* and *A. hiranoi* (strain NIES-612). They are closely related species with few differences in morphology [38,41]. *A*. *pseudogonyaulax* could be genetically distinct from *A. hiranoi* within a short distance, based on the larger partition of the LSU rDNA [25]. The D1–D3 region was also not effective in identifying *A. australiense* and *A*. *tamarense*. These species were identified as complex *A*. *tamarense* and difficult to identify based on the LSU rDNA. The phylogenetic relationship between *Alexandrium* spp. was complex based on the D1–D3 region as documented by Menezes et al. [37]. They are a polyphyletic group containing two species, *A*. *leei* and *A. diversaporum,* which belong to a clade with other genera (*Gonyaulax spinifera* and *Lingulodinium polyedra*) in the D1–D3 phylogeny (Figure 3), whereas *A*. *leei* and *A. diversaporum* belonged to a sister clade of *A*. *limii, A*. *ogatae, A*. *pseudogonyaulax,* and *A. taylorii* in the ITS phylogeny (Figure 4).

### 3.2. Effects of Temperature and Salinity on Growth of Alexandrium Species

#### 3.2.1. *Alexandrium fraterculus*

In our experiments, A. fraterculus was able to thrive at salinities between 20 psu and 35 psu and temperatures between 21 °C and 27 °C. The fastest cell division occurred at 24 °C and salinities of 25–30 psu, but cell density was highest at 27 °C within the same salinity range after 10 days. Growth was significantly reduced at salinities below 20 psu, regardless of temperature, indicating poor tolerance to low salinity [42]. The observed maximum division rates (0.2–0.4 divisions per day) aligned with those of previous studies [42]. These results suggest that blooms of A. fraterculus are likely to occur in Nha Trang Bay’s offshore waters in January–February, when the water is cool enough for optimal growth. Additionally, light and dark cycles can impact growth, potentially disrupting photosystem function and biological rhythms when exposed to continuous light [43,44].

#### 3.2.2. *Alexandrium tamiyavanichii*

The results indicate that the growth of *A*. *tamiyavanichii* is primarily influenced by temperature. This observation aligns with a previous study [45], which reported weak growth at low temperatures and a consistent maximum division rate of 0.3 divisions per day at the highest temperature tested (27 °C). In our study, growth rates increased at salinities ranging from 25 to 35 psu when temperatures exceeded 27 °C. Although growth was slightly inhibited at a salinity of 35, the findings still suggest a considerable abundance of *A*. *tamiyavanichii* at the sampling site during the warmer months from May to September. Nonetheless, further experiments are required to determine the upper temperature limit for cell growth.

#### 3.2.3. *Alexandrium leei*

*A*. *leei* grew comparatively well in most culture conditions, suggesting its ability to adapt to a wide range of salinities, ranging from 20 to 35 psu at 24 °C–27 °C. Notably, local strains can adjust their physiology to thrive in different environmental conditions [46]. Consequently, A. leei isolated from Vietnamese waters is capable of surviving and growing well at tropical temperatures ranging from 24 °C to 27 °C.

#### 3.2.4. *Alexandrium pseudogonyaulax*

At a salinity of 25 psu, the cell density doubled, with a 3 °C increase in temperature. The best cell growth was observed at a salinity of 30 psu and a temperature of 27 °C in our experiments. Previous studies have reported broad salinity (25 –35 psu) and temperature (20 –30 °C) ranges for different *A*. *pseudogonyaulax* strains [47]. Our findings indicate that the strain isolated from Nha Trang Bay can thrive in warm tropical waters almost year-round.

These autecological experiments align with studies on other *Alexandrium* species. For instance, the cell division rate of Alexandrium affine is the highest, ranging from 0.25 to 0.34 divisions per day at temperatures of 29 °C to 30 °C, with rapid cell death observed at 35 °C [23]. *Alexandrium monilatum* does not grow at 15 °C and a salinity of 15 [48]. Another study on the dynamics of *Alexandrium ostenfeldii* blooms in a coastal bay in southwest Finland revealed that this species thrives at temperatures of 17 °C, with peak cell densities reaching 30 × 10^3^ cells L⁻^1^ [49].

Notably, the growth of *Alexandrium* species can also be influenced by other abiotic and biotic factors, such as physical forces, nutrient availability, and grazing pressure [48,50]. Our experiments provide rough estimations of the optimal temperature and salinity conditions for potential bloom events in Vietnamese waters.

### 3.3. Toxins

In Vietnam, there are 17 species of *Alexandrium*, but there is a lack of knowledge on PSP toxins. *A. minutum*, which is found in shallow waters such as shrimp ponds, produces GTX1/4 [51], as well as a new GTX analog [52]. The two strains of *A. affine* and *A*. *leei* collected in Ha Long and Nha Trang bays are either non-toxic or have very low toxicity. The toxic profiles of these strains include NeoSTX, STX, and GTX1/4 [22]. *A. affine* in Honda Bay, Palawan, the Philippines, was found to produce PSTs [53]. These results are contradictory, suggesting either a misidentification of the species or the presence of both toxic and non-toxic strains. On the other hand, *A*. *fraterculus* did not produce PSTs [37]. *Alexandrium pseudogonyaulax* was reported to produce goniodomin A [54] but this species has not undergone toxicity testing in Viet Nam. However harmless blooms of this species were recorded in tiger shrimp ponds (12°24′27″–109°09′35″) north of Nha Trang city in August 2004 when the temperature was 30 °C and the salinity was 33 psu [22].

## 4. Conclusions

Seventeen species of *Alexandrium* have been identified in Vietnamese waters. In addition to morphological research, phylogenetic studies should be conducted to support accurate species identification. Among these species, *A*. *fraterculus*, *A*. *leei*, *A*. *pseudogonyaulax*, and *A*. *tamiyavanichii* exhibited similar growth patterns. These species achieved their highest growth rates at temperatures of 24 °C and 27 °C and salinities ranging from 25 psu to 35 psu.

They demonstrated tolerance to low salinity levels of 20 psu when temperatures ranged from 18 °C to 27 °C, although their growth rates gradually declined in cooler conditions. These findings suggest a significant potential for *Alexandrium* blooms in tropical coastal waters during warm months. Given their capacity to produce paralytic shellfish toxins (PSTs), these species pose a threat to public health and the safety of marine food products. Therefore, further studies on their autecology and toxin production are essential.

## 5. Materials and Methods

### 5.1. Temperature, Salinity, and Sampling

Temperature and salinity were recorded using a CTD SeaBird 19+ at a fixed station in Nha Trang Bay, at 12°10′ N and 109°18′ E from January to September 2016; measurements were taken at a maximum water depth of −50 to 0 m. Measurements could not be taken in October, November, and December due to adverse weather conditions. In June, the species *Alexandrium affine*, *A*. *fraterculus*, and *A. tamiyavanichii* were isolated from vertical haul net samples taken from the 10 m layer to the surface layer. Additionally, *A*. *leei* and *A*. *pseudogonyaulax* (strain VINVN03) were isolated from net samples collected in June 2022 at a nearshore station at a 5 m depth.

### 5.2. Cell Isolation and Maintenance

*Alexandrium* strains were cultured and maintained in a K-enriched seawater medium [55] with a salinity of 35 psu in 25 cm^2^ cell culture flasks at 26 °C. Daylight fluorescent lamps provided 50 µmol photons m^−2^ s^−1^ in a 12h:12h light–dark regime.

Morphological characteristics of *Alexandrium* species were observed under a differential interference contrast microscopy (BX51, Olympus, Tokyo, Japan) attached to a fluorescent lamp. An Olympus DP-74 digital camera (Tokyo, Japan) was used for photography. To observe the plate pattern of *Alexandrium* species, cells were stained with Calcofluor-White M2R [56]. *Alexandrium* species were identified based on the taxonomic literature [38,57].

### 5.3. DNA Extraction, Sequencing, and Phylogenetic Analysis

#### 5.3.1. DNA Extraction and Amplification

Single cells of the species *Alexandrium* were previously sequenced for nuclear rDNA, including the D1–D3 and D8–D10 domains of the LSU, as well as the ITS1-5.8S-ITS2 regions [58,59,60]. Pellets of monocultures of *Alexandrium* spp. were collected by centrifugation at 1000 g for 5 min and washed three times with deionized water. The pellets were then frozen before the addition of a 10% Chelex 100 solution (Molecular Biology Grade Resin, Bio-Rad, Hercules, CA, USA) as the extraction buffer.

DNA extraction was performed by heating the samples to 94 °C for 10 min. After extraction, the samples were stored at 4 °C. The extracted DNA was subsequently diluted tenfold before being subjected to PCR.

The D1–D3 and D8–D10 domains of the LSU and ITS1-5.8S-ITS2 rDNA regions were amplified via polymerase chain reaction (PCR) with a 25 µL mastermix comprised of TAQ buffer 1× (5×, Biolabs, Ipswich, MA, USA), dNTP 0.8 mM (VWR, Søborg, Denmark), TAQ polymerase 5 units (VWR, Denmark), 0.8 µM BSA (BioLabs, USA), the three primers (0.8 µM), and 1.5 µL of template DNA.

The PCR cycle conditions were an initial denaturation at 94 °C for 2 min and 35 cycles of the following conditions:

- Denaturation at 94 °C for 30 s;
- Annealing at 47 °C–55 °C for 45 s;
- Extension at 72 °C for 90 s.

The primers used for amplification are listed in Table 6.

Direct sequencing of the PCR product was carried out using 1ST BASE (Selangor, Malaysia) in both directions. The consensus sequences were achieved using Clone Manager 9 (Sci-Ed, Cary, NC, USA). Three strains of *Alexandrium* including *A. affine* (VINVN01), *A. fraterculus* (VINVN02), and *A*. *pseudogonyaulax* (VINVN03) were sequenced and their sequences were deposited in GenBank with the following accession numbers: PP106432, PP106433, PP106434 (ITS rDNA, ITS1-5.8S-ITS2 region); PP106429, PP106430, PP106431 (LSU rDNA, D1–D3 region); and PP106377, PP106378, PP106379 (LSU r-DNA, D8–D10 region).

#### 5.3.2. Phylogenetic and *Bioinformatic Analysis*

The three rDNA regions—D1–D3, D8–D10, and ITS1-5.8S-ITS2—were analyzed independently. Sequences from *Alexandrium* spp., *Goniodoma polyedricum*, *Gonyaulax spinifera*, *Lingulodinium polyedra, Ceratocorys horrida*, Fukuyoa yasumotoi, and *Gambierdiscus vietnamensis* (outgroup) were obtained from GenBank.

Each dataset was aligned using CLUSTAL W in MEGA X [65] and manually refined for accuracy. The *p*-distance between sequences was calculated using MEGA X [66]. Phylogenetic inference based on maximum likelihood (ML) was performed using IQ-TREE via its web server [67]. ModelFinder in IQ-TREE selected the following optimal partitioning models for ML inference based on the minimum Bayesian Information Criterion (BIC) scores: TN + F + G4 for D1–D3, TN + F + I + G4 for D8–D10, and GTR + F + G4 for ITS1-5.8S-ITS2 [68,69]. Node support was assessed with 1000 iterations of ultra-fast bootstrap (BP) analysis [70].

Bayesian inference (BI) was conducted using MrBayes v.3.2.2 [71] with the Metropolis Coupled Markov Chain Monte Carlo (MCMC) algorithm and the GTR + G model. Two parallel runs with four chains each (three heated and one cold) were executed for 1 million generations, sampling a tree every 100 generations. The resulting ML and BI trees were visualized using Figtree v.1.4.3.

### 5.4. Growth Experiments

*Alexandrium fraterculus* and *Alexandrium tamiyavanichii* were cultured under varying combinations of salinity (15, 20, 25, 30, and 35 psu) and temperature (18, 21, 24, and 27 °C). In contrast, *A*. *leei* and *A*. *pseudogonyaulax* were cultivated only at temperatures of 24 °C and 27 °C. Three replicates were prepared for each specific salinity/temperature combination using Erlenmeyer flasks containing 500 mL of K medium, with an initial cell density of approximately 180 to 200 cells mL⁻^1^.

The flasks were placed in a temperature-controlled incubator (SANYO Versatile Environmental Test Chamber MLR-352H, Gunma, Japan) under a 12 h light and 12 h dark cycle, with photosynthetically active radiation of 65 µmol photons m⁻^2^ s⁻^1^. Cell density was measured every 48 h, starting from the second day of incubation, with three replicates per culture flask. Culture experiments were conducted over 10 days for growth curve analysis and 14 days for division rate assessment.

The growth rates of the cultures were calculated using the natural logarithm [72].

$$k=\frac{Ln(\frac{N_{1}}{N_{0}})}{t}$$

where N_1_ and N_0_ are the cell densities at t_1_ and t_0_ times (day), respectively. To assess the effect of temperature and salinity on the growth of *Alexandrium* species, we first conducted a repeated-measures three-way ANOVA on growth rates, with temperature and salinity levels as between-subject factors and culture day as the within-subject factor. Since Mauchly’s test indicated that the sphericity assumption was violated, we applied the Greenhouse–Geisser correction to the ANOVA results. Since statistical significances were found among most individual factors and their mixed effects, we subsequently performed Tukey HSD post hoc tests on growth rate according to each culture day among all temperature and salinity combinations. Statistical analyses and visualization were carried out using Rstudio (version 4.3.1), using the ‘readxl’ [73], ‘car’ [74], rstatix [75], and ‘ggplot2’ packages [76].

### 5.5. Paralytic Shellfish Toxin Analysis

*Alexandrium* cells were harvested at the exponential growth phase. Algal pellets were freeze-dried (Beta 1–8 LSCbasic Martin Chris, Herrenberg, Germany). Dried algal pellets were stored in cryo vials at −20 °C for 4–6 months before analysis. Dried cell pellets were suspended in 500 µL of 0.03 M acetic acid for PSP toxin (PST) analysis. The samples were subsequently transferred into a FastPrep tube containing 0.9 g of lysing matrix D (Thermo Savant, Illkirch, France). The samples were then homogenized via reciprocal shaking at maximum speed (6.5 m s^−1^) for 45 s in a Bio101 FastPrep instrument (Thermo Savant). Toxin analysis was performed via ion-pair chromatography with post-column derivatization and fluorescence detection, as detailed in [3].

## Figures and Tables

**Table 1:** List of *Alexandrium* species from Vietnam.

Taxa	References
*Alexandrium affine* (Inoue et Fukuyo) Balech 1985	[22]
*A. compressum* (Fukuyo, Yoshida et Inoue) Balech 1995	[22]
*A. concavum* (Gaarder) Balech, emend. Nguyen-Ngoc et Larsen 2004	[22]
*A*. *foedum* Balech 1990	[21]
*A. fraterculus* (Balech) Balech 1985,	[22]
*A. gaarderae* Nguyen-Ngoc et Larsen nom. nov. 2004	[22]
*A. globosum* Nguyen-Ngoc et Larsen sp. nov 2004	[22]
*A. insuetum* Balech 1985	[22]
*A. kutnerae* (Balech) Balech 1985	[22]
*A*. *leei* Balech 1985,	[22]
*A. minutum* Halim 1960	[22]
*A. ostenfeldii* (Paulsen) Balech et Tangen 1985	[22]
*A*. *pseudogonyaulax* (Biecheler) Horiguchi ex Yuki et Fukuyo 1992	[22]
*A*. *satoanum* Balech 1992	[19]
*A*. *tamarense* (Lebour) Balech 1995	[22]
*A*. *tamiyavanichii* Balech 1994,	[22]
*A*. *tamutum* M.Montresor, A.Beran and U.John 2004	[19,20]

**Table 2:** Alignment details generated for the D1–D3 and D8–D10 domains of the LSU and ITS1-5.8S-ITS2 rRNA regions.

Alignment	Length (bp)	Conserved Sites	Variable Sites	Parsim-Infor Sites	Singleton Sites
D1–D3	1136	296	754	507	228
D8–D10	954	688	260	138	122
ITS–5.8S–ITS2	641	104	524	434	90

**Table 3:** Growth rate of *Alexandrium leei* at 24 °C and 27 °C.

	N_1_/N_0_	N2/N1	N3/N2	N4/N3	N5/N4	N6/N5	N7/N6	N8/N7	N9/N8
Salinity	Temperature 24 °C								
15	0.28	0.04	0.20	0.43	0.58	0.40	0.58	0.36	−0.17
20	0.42	0.19	0.13	1.06	0.02	0.36	0.64	0.36	0.48
25	−0.01	0.21	0.56	0.76	0.41	0.66	0.44	0.28	0.19
30	−0.06	0.49	0.56	0.55	0.22	0.80	0.35	0.40	0.12
35	0.29	0.14	0.47	0.49	0.45	0.57	0.47	−0.13	0.50
	Temperature 27 °C								
15	−0.16	0.85	0.65	0.63	0.46	0.31	0.31	0.06	−0.27
20	0.39	0.65	0.80	0.42	0.50	0.56	0.33	0.15	−0.75
25	0.55	0.68	0.61	0.55	0.35	0.55	0.36	0.09	−1.20
30	0.53	0.66	0.82	0.60	0.27	0.64	0.06	0.11	−1.07
35	0.51	0.87	0.65	0.48	0.37	0.59	0.28	0.12	−0.63

**Table 4:** Growth rate of *Alexandrium pseudogonyaulax* at 24 °C and 27 °C.

	N_1_/N_0_	N2/N1	N3/N2	N4/N3	N5/N4	N6/N5	N7/N6	N8/N7	N9/N8
Salinity	Temperature 24 °C								
15	−0.05	0.02	−0.59	−0.21	−0.18	−0.18	−0.41	−0.40	−0.72
20	0.30	0.15	−0.04	0.41	0.18	0.24	0.25	−0.07	−0.27
25	0.31	0.04	0.25	0.50	0.15	0.15	0.29	0.12	−0.04
30	0.29	0.07	−0.01	0.03	−0.01	0.12	0.01	0.10	0.02
35	0.27	−0.03-	0.05	0.09	0.04	0.10	0.04	−0.01	−0.08
	Temperature 27 °C								
15	0.07	0.19	−0.02	−0.38	−0.38	−0.50	−0.41	−2.42	0.54
20	0.14	0.59	0.07	0.14	0.27	0.15	0.24	0.09	−0.07
25	0.01	0.97	0.16	0.37	0.32	0.11	0.57	0.11	0.21
30	0.39	0.23	0.36	0.86	0.30	0.10	0.16	0.13	0.19
35	0.16	0.45	0.38	0.21	0.86	0.12	0.08	0.07	0.03

**Table 5:** Detected limits of PSTs.

Toxins	LODs (Limits of Detection)	
	E276-3 (fg/Cell)	E276-4 (fg/Cell)
C1/C2	1.58	0.98
GTX 4	11.27	6.97
GTX 1	15.46	9.56
dc GTX 2	0.85	0.52
dc GTX 3	0.73	0.45
GTX 2	0.95	0.59
GTX5	3.12	1.93
GTX3	1.02	0.63
Neo STX	9.61	5.95
dcSTX	1.73	1.07
STX	1.41	0.87

**Table 6:** Primers used for the DNA sequencings of *Alexandrium* rDNA.

Target	Primer	Nucleotide Sequence (5′ to 3′)	Reference
D1–D3	D1R	ACCCGCTGAATTTAAGCATA	[61]
	D3B	TCGGAGGGAACCAGCTACTA	[61]
D8–D10	FD8	GGATTGGCTCTGAGGGTTGGG	[62]
	RB	GATAGGAAGAGCCGACATCGA	[62]
	677R	TGTGCCGCCCCAGCCAAACT	[63]
	421F	ACAGCCAAG GGA ACGGGCTT	[63]
ITS–5.8S–ITS2	ITSA	GTAACAAGGTHTCCGTAGGT	[64]
	ITSB	AKATGCTTAARTTCAGCRGG	[64]

## Data Availability

The original contributions presented in this study are included in the article. Further inquiries can be directed to the corresponding authors.

## Supplementary Materials

The following supporting information can be downloaded at: https://www.mdpi.com/article/10.3390/toxins17020081/s1, Figure S1: Monthly temperature and salinity profiles of sampling station; Figure S2: Growth rate of *A*. *fraterculus* at different salinities and temperatures; Figure S3: Growth rate of *A*. *tamiyavanichii* at different salinities and temperatures; and Video S1: *A*. *tamiyavanichii* in culture with long chains of up to 32 cells.
