# Supplementary material for: Phylogenetic and Autecology Characteristics of Five Potentially Harmful Dinoflagellate Alexandrium Species (Dinophyceae, Gonyaulacales, Pyrocystaceae) in Tropical Waters: A. affine, A. fraterculus, A. leei, A. pseudogonyaulax, and A. tamiyavanichii
Source: Toxins (Basel). 2025 Feb 10;17(2):81. doi: 10.3390/toxins17020081 (PMC11862000; doi:10.3390/toxins17020081)
Supplement: Supplementary file 1 [file toxins-17-00081-s001.zip › toxins-3390038-supplementary.pdf]

Supplementary Materials: Phylogenetic and Autecology Characteristics of Five Potentially Harmful Dinoflagellate *Alexandrium* species (Dinophyceae, Gonyaulacales, Pyrocystaceae) in Tropical Waters: *A. affine, A. fraterculus, A. leei, A. pseudogonyaulax,* and *A. tamiyavanichii*

Lam Nguyen-Ngoc, Dang-Minh L., H. Doan-Nhu, H. M. Pham, B. Krock, N. D. Huynh-Thi, L. V. Tran-Thi, M. H. Tran-Thi, Anh H. Pham, V. Nguyen-Tam, T. T. Nhan-Luu and H. H. Do

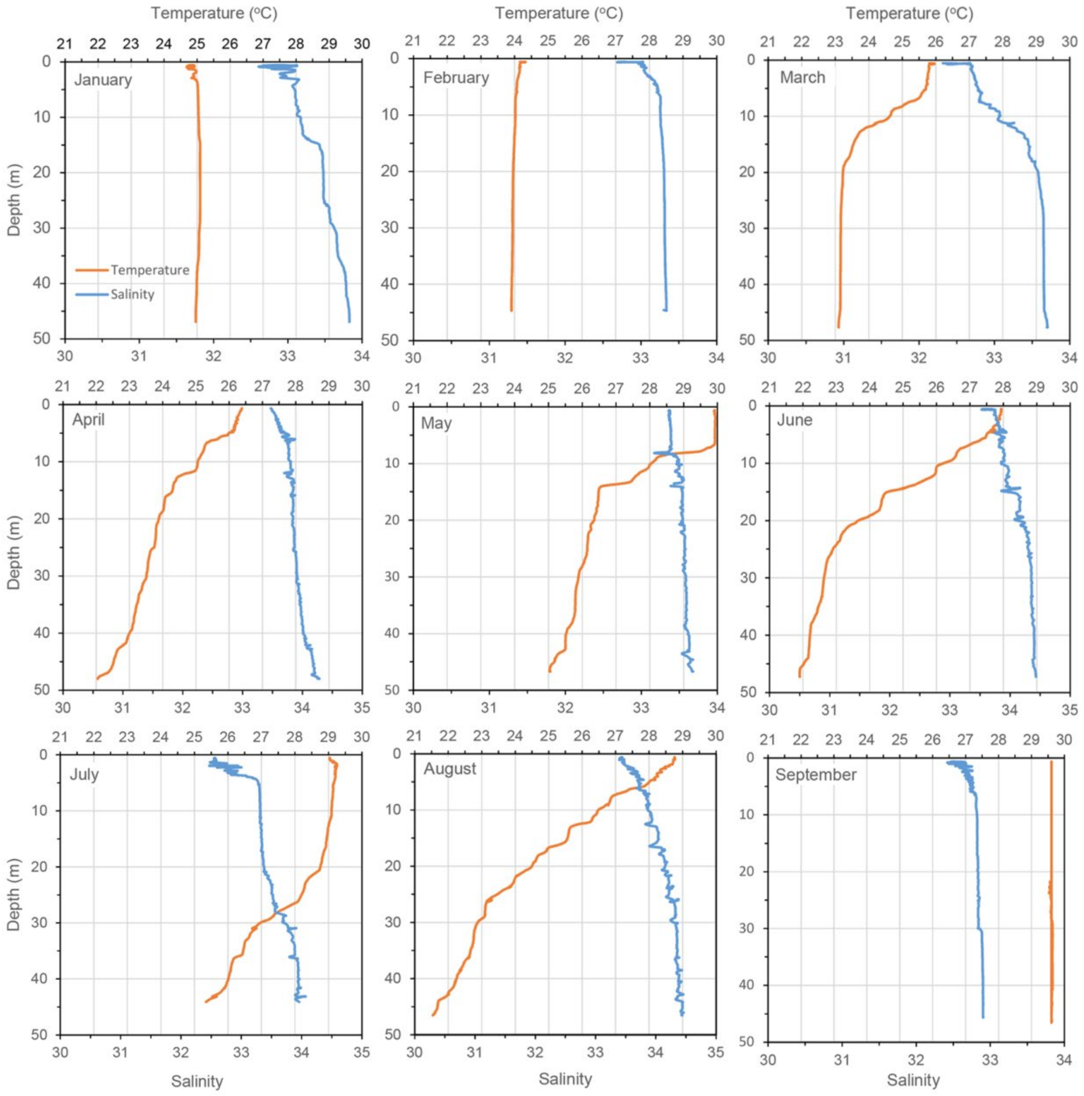

Figure S1. Monthly temperature and salinity profiles of sampling station.

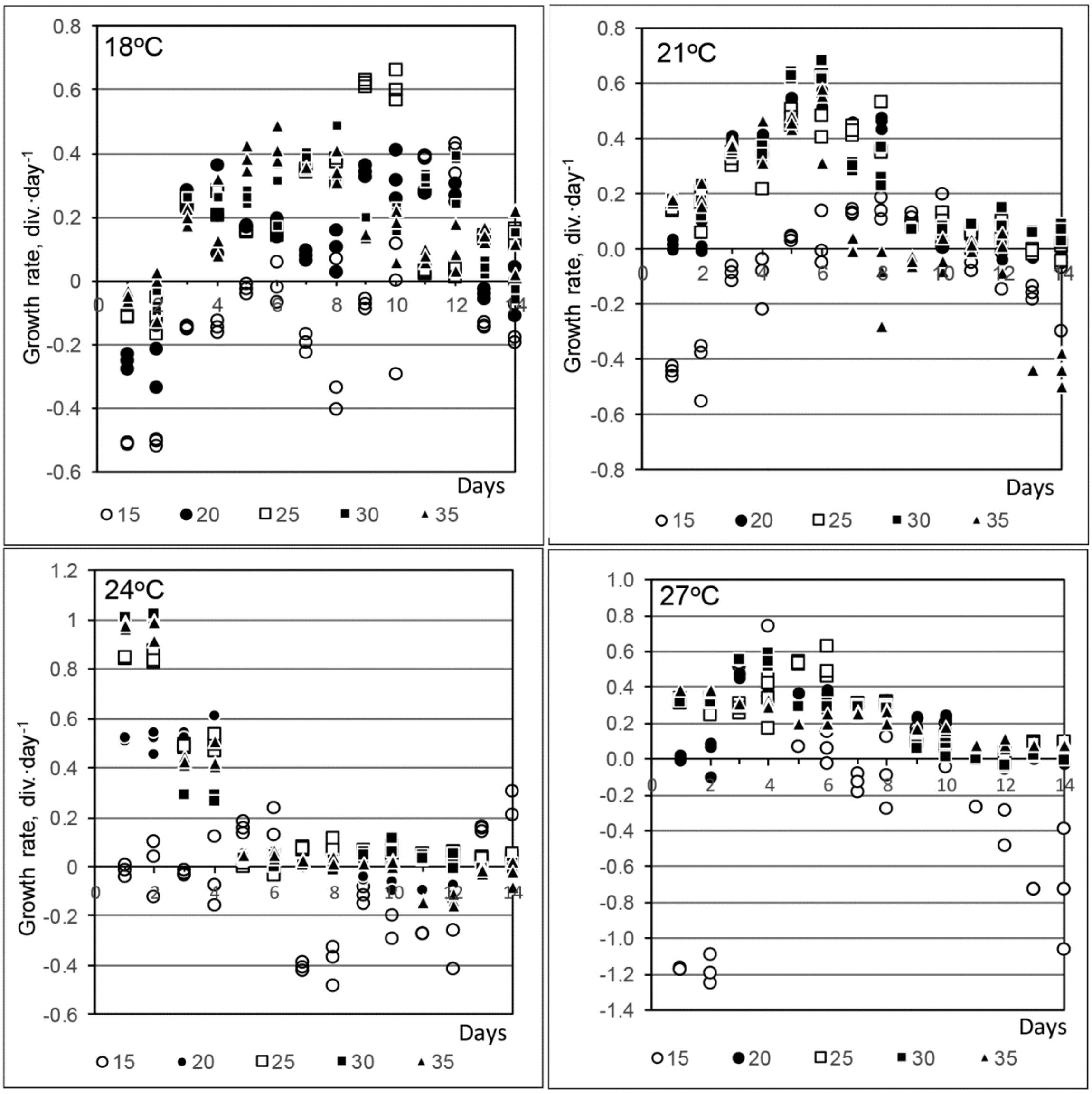

Figure S2. Growth rate of *A. fraterculus* at different salinities and temperatures.

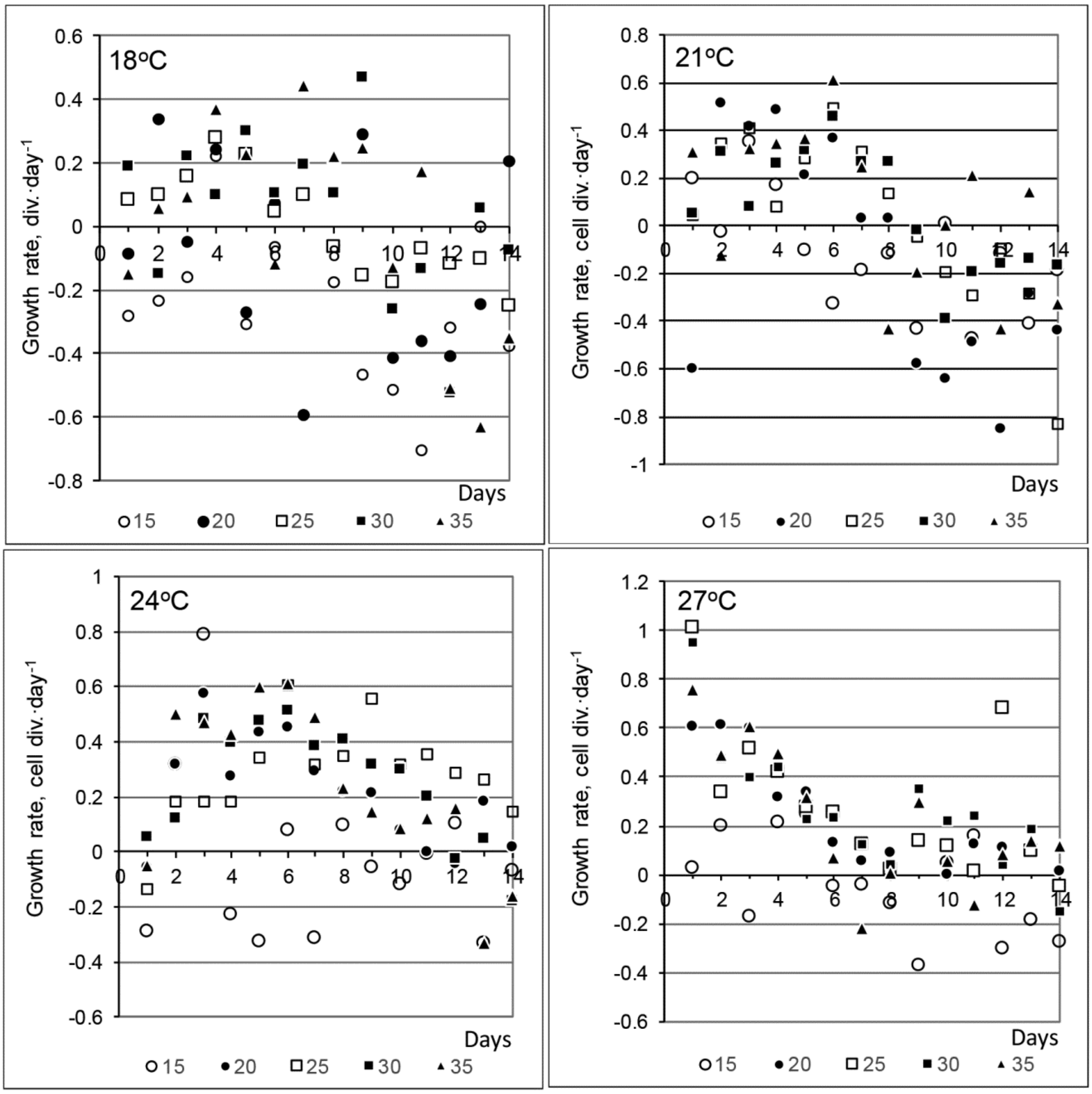

Figure S3. Growth rate of *A. tamiyavanichii* at different salinities and temperatures.
